# How important is the physician in an era of digitalization and alternative information sources? A survey among parents of children with developmental disorders

**DOI:** 10.1007/s00431-025-06222-5

**Published:** 2025-06-04

**Authors:** Charlyn Kreis, Martina P. Neininger, Teresa Vela Martin, Gudrun E. Krause, Thilo Bertsche, Astrid Bertsche, Sarah Jeschke

**Affiliations:** 1 Department of Neuropediatrics, Hospital for Children and Adolescents, University Medicine Greifswald, 17475 Greifswald, Germany; 2 Department of Neuropediatrics, Hospital for Children and Adolescents, University Medicine Rostock, 18057 Rostock, Germany; 3 German Center for Child and Adolescent Health (DZKJ), Partner Site Greifswald/Rostock, 17475 Greifswald, Germany; 4 Drug Safety Center and Clinical Pharmacy, Institute of Pharmacy, Medical Faculty, Leipzig University, 04103 Leipzig, Germany; 5 Department of Psychiatry and Psychotherapy, Leipzig University Hospital, 04103 Leipzig, Germany; 6 Social Pediatric Center, Hospital for Children and Adolescents, University Medicine Rostock, 18057 Rostock, Germany

**Keywords:** Developmental disorders, Parents, Physician, Source of information, Handling a diagnosis, Acceptance

## Abstract

A developmental disorder can lead to major issues in daily life of affected families. As physicians’ time is limited, parents often look for alternative sources of information. We aimed at investigating parents’ preferences and judgement regarding different sources of information. At the neuropediatric outpatient department of a university hospital, we conducted interviews with parents of children with a developmental disorder. Altogether, 83 parents took part in the interview. At the time of the interview, 12/83 (14%) of the parents lacked confidence in managing their child’s diagnosis in daily life, of whom 10/12 (83%) had the diagnosis for one year or more. Parents most frequently received information about the diagnosis from the physician (48/83; 58%), from the internet (22/83; 27%), or from the early intervention center (6/83; 7%). They judged the physician (32/83; 39%), the early intervention center (16/83; 19%) and the internet (12/83; 14%) as most helpful for handling their child’s diagnosis.

* Conclusion*: The physician is still the most important source of information for parents of children with a developmental disorder. The internet and early intervention centers are also crucial for parents to gain information about the condition and handling it in daily life.

**Table:** 

**What is known:** • *Developmental disorders can have a major impact on the life of affected families*. • *Parents can be supported in accepting the condition by various players of the healthcare system*.	
**What is new:** • *For parents, the physician plays a crucial role in dealing with the condition in daily life*. • *Some parents consult the internet and early intervention centers more often than the physician*.

**Supplementary Information:**

The online version contains supplementary material available at 10.1007/s00431-025-06222-5.

## Introduction

Developmental disorders can have a major impact on the life of affected families requiring support for these families [1]. However, physicians’ capacities are limited. Thus, other players of the healthcare system such as early intervention centers or the internet can be valuable resources. To date, it remains unclear how important the physician is as a source of information in times of digitalization and other players in the healthcare system. We therefore examined the parents’ preferences of different sources of information as well as the perceived helpfulness of those sources in handling and accepting the diagnosis.

## Materials and methods

After ethics approval (A 2021–0250), this prospective study was conducted from December 2021 to May 2022. A member of the study team approached parents or foster parents of children with a developmental disorder, i.e. mixed specific developmental disorder (ICD 10: F83) in children aged younger than 6 years, and in children aged six years or older learning disorder (ICD 10: F81.9) or intellectual disability (ICD 10: F70, F71, F72 and F73), who had an appointment at the neuropediatric outpatient department of a university hospital and invited them to take part in a semi-structured interview. Adequate German language skills to understand and answer the questions were necessary to be included into the study. Written informed consent was obtained from all parents. If both parents were present, we interviewed the one who stated to be more in charge of the child’s care.

A study team including a neuropediatrician, a child and adolescent psychotherapist, a pharmacist experienced in pediatric pharmaceutical care, and a medical student developed the interview. We piloted the questionnaire with five parents who were also included in the main study. Based on the pilot survey, comprehensibility of the questionnaire was improved, particularly language was simplified. The final questionnaire consisted of 4 questions on parental confidence in handling the developmental disorder and information seeking behavior (Online Resource 1). For 2 questions, parents were also asked to rank given items (physician, internet, other parents, brochures, social media, self-help groups/forums, and early intervention centers). Furthermore, sociodemographic data were retrieved from the patients’ medical records and the questionnaire. To minimize potential interviewer effects, the medical student of the study team conducted all interviews. Interview duration was approximately 10 min and all interviews took place at the neuropediatric outpatient department.

Data analyses were performed with Microsoft® Excel 2021 (Version 16.63.1) and SPSS (V29.0.0.0). To express the ranking of prioritization, the study team calculated a score (S) for the first 3 items reflecting the prioritization and relative frequency of the respective item: S = 3n_1_ + 2n_2_ + n_3_, as published previously. In this context, n_1/2/3_ indicates the count of nominations of the respective item at ranking position 1/2/3 [2]. We did not perform statistical corrections such as weighting of items.

We conducted an exploratory data analysis. To investigate whether the parameters type of child’s developmental disorder, child’s age, mother’s age, father’s age, or who participated in the interview may have an influence on the rating of frequency of use and perceived helpfulness of physician and internet, we performed Kruskal–Wallis tests or calculated Spearman’s ρ, depending on the underlying data. We also investigated a potential correlation between the ranking of frequency of use and perceived helpfulness by calculating Kendall’s τ-b. A p-value ≤ 0.05 was considered to indicate significance. Effect sizes were determined using Cohen’s classification [3]. To enhance readability, we only report significant results.

## Results

A total of 88 parents were invited to take part in this survey. Three parents were excluded because they did not understand the questions properly, and 2 parents declined to participate. Sociodemographic data are displayed in Table 1.

**Table 1:** Characteristics of participants and patients

Characteristics	Values
Total of parents [*n*]	83
Gender: female; male [*n* (%)]	66 (80%); 17 (21%)
Median age of parent (Q25/Q75; min/max) [years]	37 (33.25/40.75; 22/61)
Current occupation [*n* (%)]	
Sales, services, trade, technology and industry	32 (39%)
Health and nursing professions	21 (25%)
Office and administration	9 (11%)
Educational/pedagogical professions	5 (6%)
Currently not/not anymore/not yet employed	16 (19%)
Gender of patients	
female; male [*n* (%)]	43 (51%); 40 (49%)
Median age of patients (Q25/Q75; min/max) [years]	5.4 (3.9/8.4; 0.8/17.7)
Age at the time of diagnosis (Q25/Q75; min/max) [years]	1.9 (0.25/3; 0/12.7)
Time span between diagnosis and interview (Q25/Q75; min/max) [years]	3.9 (2/6; 0.1/17.7)
Diagnosis [*n* (%)]	
Intellectual disability	29/83 (35%)
Learning disability	6/83 (7%)
Mixed-specific developmental disorder	48/83 (58%)

When asked how long it took parents to feel confident in dealing with the specific symptoms and resulting behavior of their child’s developmental disorder in their daily lives, 22/83 (27%) of respondents stated that this happened immediately after receiving the diagnosis, 29/83 (35%) answered they needed less than 6 months, and 11/83 (13%) needed more than 6 months but less than 1 year. Of the parents, 12/83 (14%) did not feel confident at the time of the study, of whom 10/12 (83%) had the diagnosis for 1 year or more.

**Fig. 1:**
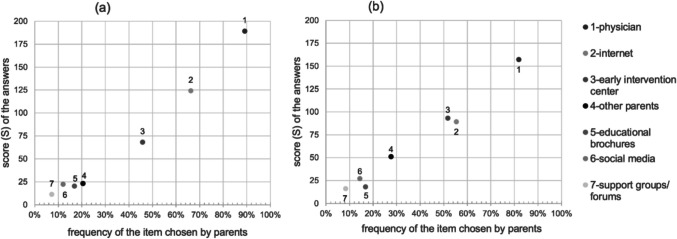
Frequency and prioritization of persons, institutions or resources that (**a**) families used most frequently to get information about the child’s diagnosis and (**b**) helped them most with handling the diagnosis in daily life (ranking list with 7 items); The score (S) was calculated from the parents’ prioritization and frequency of each selectable item: S = 3n_1_ + 2n_2_ + n_3_; in this context, n_1/2/3_ indicates the count of nominations of the respective item at ranking 1/2/3. For example, 55/83 (66%) parents used the internet as a source of information, of whom 22 ranked it first, 25 second and 8 third, resulting in S = 124

Regarding the source of information parents used most frequently to get information about their child’s diagnosis, most parents selected the physician (48/83, 58%) as their first choice. The frequency and prioritization of this item and the other ones are shown in Fig. 1a.

Regarding the source of information the parents considered most helpful for handling their child’s condition, most parents selected the physician (32/83, 39%) as their first choice, followed by early intervention centers (16/83, 19%), and the internet (12/83, 14%; Fig. 1b).

Regarding the rankings of the physician and the internet, we found correlations between the ranking of use of frequency and perceived helpfulness (physician: Kendall’s τ-b 0.540, p < 0.001, Cohen’s effect classification: large; internet: Kendall’s τ-b 0.328, p = 0.007, Cohen’s effect classification: medium). Regarding the ranking of the frequency of use of the internet, we found a correlation with the parent’s age, the higher the age, the higher the ranking (mother’s age: Spearman’s ρ 0.349, p = 0.007, Cohen’s effect classification: medium; father’s age: Spearman’s ρ 0.294, p = 0.026, Cohen’s effect classification: small).

## Discussion

### Lack of confidence

Most parents reported feeling confident in handling their child’s developmental disorder within 1 year after receiving the diagnosis. However, 14% of parents did not feel confident at the time of the interview, with 83% of those parents having had to deal with the diagnosis for over a year. One possible reason could be insufficient knowledge about the child’s developmental disorder [4]. Limited confidence in dealing with the diagnosis may also result from a lack of acceptance of the developmental disorder, or from challenges that arise in the course of the disease, e.g., higher care requirements for older children or subsequent impairments. In addition, other risk factors such as comorbid disorders or chronic stress can complicate the adaptation process [4]. Therefore, it is crucial to identify parents with greater support needs to establish interventions.

### Most frequently used sources of information

The physician remained the most frequently used source of information. The internet was also widely used as a source of information and, for a quarter of the parents, it was even the one most frequently used. Many parents rely on the internet uncritically and thus alter healthcare decisions made with their physician [5]. Particularly in cases of severe disease, another study showed that parents were more likely to use non-evidence-based measures, which may conflict with the treatment plan or remain undisclosed to the physician [6]. This should be considered in medical consultations and may need to be discussed with the parents. Therefore, a trust-based relationship with the physician is crucial [7]. At this point, talking about online medical information during medical consultations and thereby sharing the responsibility for knowledge can help to strengthen the parent-physician relationship and improve decision-making [8]. In Germany, so-called digital health applications facilitate the access to verified digital medical information and support the individual patient in the treatment process [9]. These applications are certified by the regulatory authorities and can be prescribed by physicians, which may contribute to strengthen the patient-physician relationship. However, there are currently no digital health applications available to support families of children with developmental disorders. Early intervention centers were also frequently used to obtain information. Accordingly, employees are required to have a high level of expertise to provide accurate information to parents. Other sources of information, such as brochures, other parents, support groups, and social media were used less frequently. The low usage of brochures might be explained by digitalization. The other sources may serve a different purpose, such as providing emotional support [10].

### Most helpful source for handling the condition

For parents, the physician was most helpful in managing the child’s developmental disorder in daily life. Even early intervention centers, where parents and their children often have weekly contacts, were not ranked as high as the physician. This may be caused by the fact that parents expect the physician to provide them with all-round care [11]. The physician was more frequently placed at the top of the ranking compared to the internet. One reason might be that real people allow a mutual exchange of information, while the internet only provides information. However, this may change in the future with the implementation of artificial intelligence-assisted systems. Those systems are trained in responding in a validating manner and are able to provide specific recommendations. Previous studies indicated a positive effect on people with chronic conditions [12]. Other parents were identified as a helpful source for handling the condition in daily life, as they can offer emotional support as well [10]. Brochures and self-help groups were less often highly ranked by parents, possibly due to a lack of suitable offers or a lack of time. Surprisingly, social media also played a secondary role. This could be explained by a lack of suitable content or because parents may use social media for other topics.

## Limitations

The study design allows statements about frequencies and prioritization. However, it was not possible to quantify how much more a source was used compared to others. Due to the exploratory character of the study, conclusions on correlations have to be drawn with caution. As the study was performed in 1 hospital in Germany, results should be interpreted with care and generalizability may be limited.

## Conclusion

The physician still plays a key role for parents of children with developmental disorders. The internet or early intervention centers are also indispensable in today’s treatment, which is why they have to be taken into account in treatment.

## Supplementary Information

Below is the link to the electronic supplementary material.

ESM 1 (PDF 116 KB)

## Data Availability

The data that support the findings of this study are not openly available due to reasons of sensitivity and are available from the corresponding author upon reasonable request.
