# Supplementary material for: How important is the physician in an era of digitalization and alternative information sources? A survey among parents of children with developmental disorders
Source: Eur J Pediatr. 2025 Jun 4;184(7):391. doi: 10.1007/s00431-025-06222-5 (PMC12137483; doi:10.1007/s00431-025-06222-5)
Supplement: Supplementary file 1 — ESM 1 (PDF 116 KB) [file 431_2025_6222_MOESM1_ESM.pdf]

How important is the physician in an era of digitalization and alternative information sources? A survey among parents of children with developmental disorders

European Journal of Pediatrics

Charlyn Kreis[1,2,3], Martina P. Neininger[1,3,4], Teresa Vela Martin[1,2,3], Gudrun E. Krause[5], Thilo Bertsche[4], Astrid Bertsche[1,2,3*], Sarah Jeschke[1,2,3]

Authors' affiliation addresses:

[1]Department of Neuropediatrics, Hospital for Children and Adolescents, University Medicine Greifswald, 17475 Greifswald, Germany

[2]Department of Neuropediatrics, Hospital for Children and Adolescents, University Medicine Rostock, 18057 Rostock, Germany

[3]German Center for Child and Adolescent Health (DZKJ), partner site Greifswald/Rostock, 17475 Greifswald, Germany

[4]Drug Safety Center and Clinical Pharmacy, Institute of Pharmacy, Medical Faculty, Leipzig University, 04103 Leipzig, Germany

[5]Social Pediatric Center, Hospital for Children and Adolescents, University Medicine Rostock, 18057 Rostock, Germany

***Corresponding author:**

Prof. Dr. med. Astrid Bertsche, University Medicine Greifswald, Hospital for Children and Adolescents, Department of Neuropediatrics, Ferdinand-Sauerbruch-Str. 1, 17475 Greifswald, Germany, Tel.: +49/3834/866390, Fax: +49/3834/867359, Email: astrid.bertsche@uni-greifswald.de

Questionnaire

Part A: Questions on parental confidence in handling the developmental disorder and parental information seeking behavior

1. When did your child get the diagnosis of a developmental disorder?
2. When did you feel confident in handling the specific symptoms and the behavior of your child in daily life?
   To answer this question, the parents were presented with a timeline. They could mark their answer there:

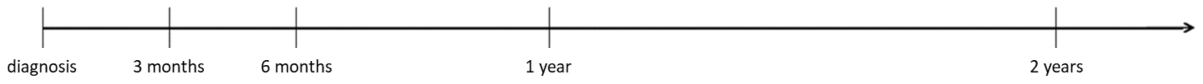

3. Which of the following sources of information did you use most frequently to get information about your child's diagnosis? Please chose from given items and rank your answers (Sources of information: Physician, internet, educational brochures, other parents, support groups/ forums, social media, early intervention center).
4. Which of the following source of information did you consider most helpful regarding the handling of your child's condition? Please choose from given items and rank your answers (Sources of information: Physician, internet, educational brochures, other parents, support groups/ forums, social media, early intervention center).

To answer questions 3 and 4, parents were given cards corresponding to each item. The parents selected the cards that applied to them and put these cards in the order of their personal prioritization.

Part B: Sociodemographic data

1. Age of parent
2. Gender of parent
3. Current occupation of parent
